# Obstetrics

**Published:** 1876-02

**Authors:** 


### V. Obstetrics.

1. *A Case in which the Fœtal Heart was heard early in Pregnancy.* Underhill. (*The Obstetrical Journal of Great Britain and Ireland*, November, 1875.)

The reporter of this case says he first visited the patient while she was suffering from typhus fever, on 6th September, 1874. A thin, lax abdominal wall readily enabled him to detect, by palpation, the outline of the enlarged uterus. With the stethoscope he "distinctly heard the double pulsation of the fœtal heart," "beating at the rate of between 160 and 170 pulsations per minute, while the mother's pulse was only about 120." A fortnight in the hospital sufficed to cure her of her fever. "She was delivered of a very large and healthy child, the writer says, "on the 8th of March, 1875, that is to say, six calendar months and two days, or 183 days, *after* I heard the fœtal heart, which was, taking 278 days as the average duration of pregnancy, only 96 days (13 weeks and 5 days) after the supposed successful coitus."

2. *Gestation continued Three Months after Rupture of Membranes, and Labor Pains lasting Thirty-six Hours.* Dr. N. J. Rains. (*American Med. Weekly*, Nov. 27, 1875.)

The subject of this observation was hoeing in her garden, 5th of June last, when she felt something "break and give way," as she expressed it, and about two gallons of water, in a few minutes, escaped. Labor pains soon set in and continued thirty-six hours, when they spontaneously subsided, at which time Dr. Rains saw her. He soon left her, with instructions to be recalled upon the return of the pains, which he supposed would soon set in again. He was recalled upon the return of the pains, which did not come on again, however, until three months had elapsed; and he then delivered her of a large, healthy child, after an easy labor.

3. *Sloughing of Uterus and Bladder as a Sequel of Labor.* Emmet. (*Med. and Surg. Reporter*, January, 1876.)

The following case is reported by Dr. Emmet, from the New York Woman's Hospital:

At the age of 22 the patient was taken in labor with her first child, and about forty-eight hours after, the waters ruptured, and a child weighing fourteen pounds was delivered by means of the forceps. The condition of the patient was favorable for two weeks, when she noticed that her urine dribbled away by the vagina. Two weeks afterward, or one month after labor, she passed some large sloughs. These sloughs were preserved by her physician, and, on examination, proved to consist of two masses—one of them consisting of the placenta, and the other the base of the bladder and cervix of the uterus.

At this point the main interest of the case rests, and, unfortunately, it is involved in obscurity. The point referred to is this: Under what circumstances did the accoucheur, after delivering a child of fourteen pounds, neglect to see that the placenta was delivered? The only explanation possible, without gravely impeaching the competency of the attendant, is to suppose that the cord, with a portion of the placenta, came away, and, from causes not explained, the deficiency was overlooked. Another question to be considered is, did the remaining placenta, directly or indirectly, cause the extensive sloughing of the pelvic viscera? This is another question difficult of solution, though, from the time intervening between the labor and the passage of the sloughs—one month—it is fair to assume that the retained placenta did have an influence. When the patient entered the hospital, nearly five months had elapsed, and her condition then was as follows: On placing the patient on her back and separating the labia, a prolapsed fundus of the bladder was discovered. When this was replaced by a sponge probang, no sign of uterus could be discovered. When she was examined by the rectum, a small nodule was discoverable in the place that should be occupied by the uterus. After a careful examination of the case it was decided that no operation for the closure of the base of the bladder could be successful, from the fact that there was not a sufficiency of tissue. It was then decided, with the consent of the patient, to perform an operation for the closing of the vagina. This was done, and after it was performed the patient enjoyed comparative comfort.

4. *The Management of Abortion.* Dr. W. D. Hazzard. (*Virginia Med. Monthly*, December, 1875.)

On being called to a case threatened with abortion, my invariable rule of practice is first to ascertain as nearly as possible the stage of the pregnancy, frequency and the extent of the pains, the amount of hæmorrhage, and the cause, if known, that has induced the symptoms present—whether they be habitual, or accidental, immediately predisposing or exciting. Having elicited all the information I can from the patient or her friends, I then institute an examination per vaginam. If I find the os uteri dilated and flaccid, with expulsive pain at the menstrual periods, whether attended with hæmorrhage or not—if the os be dilated to any considerable size, I regard the case as of doubtful character and make my prognosis accordingly. But if I find the os uteri firm and undilated, even if I find hæmorrhage attended with severe pain, I regard the case more favorably; and if the other symptoms warrant the belief that the fœtus be living, I make the effort to save the conception. I endeavor to bring my patient as speedily under the influence of opium as possible. Having done this, I endeavor to keep her under the influence of the drug as long as any symptoms remain of pain or hæmorrhage—experience having taught me that many cases may be carried to a favorable termination that under a more temporizing treatment would result in miscarriage. I am thoroughly convinced that many cases of abortion are caused by nervous irritability of the uterus, resulting in an intolerance to the presence of the fœtus in the womb; hence nature endeavors to rid the organ of its contents by expulsive efforts. If this irritability be overcome by the free and full administration of the sedatives and narcotics, the pregnancy may oftentimes be saved.

I do not think authorities have laid stress enough on the importance of thorough investigations in such cases as I have spoken of, to enable the practitioner to make a clear diagnosis. Neither do I think they attach sufficient importance to the wonderful powers of opium, administered with the view of procuring its full effect. In my hands, it has seldom disappointed my expectations.

5. *A Case of Prolonged Gestation.* PROF. FRANK WELLS. (*Bost. Med. and Surg. Jour.; Obstet. Jour.*, Dec., 1875.)

Mrs. M., a lady of great intelligence, had sexual intercourse with her husband on August 27, 1874, two days after the completion of her monthly period. On the following day her husband was called away by business, which detained him from home until the existence of pregnancy had declared itself to his wife by the cessation of the catamenia. She quickened in the early part of January, 1875, and naturally expected to be confined about the 3rd of June, for which period she engaged her nurse. Labor did not come on, however, until June 26th, nor was it completed until the following day, exactly three hundred and four days from the date of sexual congress.

The birth, which was a tedious one, necessitating the application of the forceps, was chiefly characterized by the almost entire absence of liquor annii, only sufficient being discharged to make upon the sheet a stain of the size of a silver dollar. The child, which weighed eight and one-half pounds, looked as though it had been, in a measure, macerated in the amniotic fluid, its skin being loose and wrinkled, and the epidermis peeling off in strips. At this date (November, 1875), the child (a boy) is healthy and vigorous, and weighs eighteen and one-half pounds.

The case is remarkable, not only for the long continuance of the gestation, but also for the undoubted evidences of the absorption of some, at least, of the liquor annii through the cutaneous surface of the fœtus. That this must have been the case is indicated by the fact that as late as the eighth month the child was quite freely movable in the uterine cavity, which shows that at this time there must have been some fluid in which it could float; and still further, by the absence, during gestation, of all painful motions of the child, which would undoubtedly have been felt, had the solid contents of the womb come in direct apposition to the uterine walls, without the medium of a protecting fluid.

